# Synchronous invasive ductal and invasive lobular carcinomas in contralateral breasts: a dual-pathology case report

**DOI:** 10.1097/RC9.0000000000001044

**Published:** 2026-09-29

**Authors:** Reza Hosseinpour, Taha Negahdari, Sepehr Moradi, Mahdokht Azizi, Maral Mokhtari

**Affiliations:** a Department of Surgery, Breast Surgery Division (Breast Surgery Fellowship), Yasuj University of Medical Sciences, Shahid Jalil Hospital, Yasuj, Iran; b Department of Medicine, Yasuj University of Medical Sciences, Yasuj, Iran; c General Surgery Department, Shiraz University of Medical Sciences, Fars, Shiraz, Iran; d Department of Pathology, Yasuj University of Medical Sciences, Yasuj, Iran; e Hematopathology, Pathology, Shiraz University of Medical Sciences, Shiraz, Iran

**Keywords:** breast cancer, invasive ductal carcinoma, invasive lobular carcinoma

## Abstract

**Background:**

Breast cancer remains the most commonly diagnosed malignancy among women worldwide, whereas bilateral breast cancer is an uncommon entity that presents distinct diagnostic and therapeutic challenges. Synchronous bilateral tumors with different histopathologic subtypes are particularly rare and underscore the heterogeneity of breast cancer. This report describes a case of synchronous bilateral breast cancer with invasive ductal carcinoma (IDC) and invasive lobular carcinoma (ILC) in opposite breasts.

**Case presentation:**

A 57-year-old woman presented with a palpable right breast mass. Imaging and biopsy revealed an IDC in the right breast, while further evaluation identified an occult contralateral lesion, which was confirmed as ILC. The patient underwent bilateral breast-conserving surgery with axillary dissection. Histopathology demonstrated distinct tumors with different receptor profiles. Adjuvant radiotherapy was initiated, and the patient remains symptom-free.

**Discussion:**

This case highlights the importance of comprehensive bilateral imaging and independent pathological assessment in patients with breast cancer. The coexistence of IDC and ILC illustrates biological heterogeneity and has direct implications for diagnostic evaluation, surgical planning, and adjuvant treatment strategies.

**Conclusion:**

This case underscores the importance of comprehensive bilateral imaging and individualized multidisciplinary management in synchronous bilateral breast cancer with discordant histology.

## Introduction

In 2022, breast cancer was diagnosed in approximately 2.3 million women worldwide and accounted for an estimated 670 000 deaths globally^[^1^]^. Bilateral breast cancer is relatively rare, with reported incidence rates ranging from approximately 1.4–11.8% among patients with breast cancer^[^2^]^. Bilateral breast cancer poses diagnostic and therapeutic challenges because of its rarity and the need to individualize management for each tumor^[^3^]^. This case report aims to highlight the diagnostic and therapeutic implications of synchronous bilateral breast cancer with distinct histopathologic subtypes, emphasizing the importance of comprehensive imaging and individualized management. This case report has been prepared and presented in accordance with the SCARE 2025 guidelines^[^4^]^.

HIGHLIGHTS

- Synchronous bilateral breast cancer with discordant histology is a rare clinical entity.
- Comprehensive bilateral imaging enabled the detection of an occult contralateral malignancy.
- Independent pathological assessment confirmed invasive ductal carcinoma and invasive lobular carcinoma in opposite breasts.
- Individualized, multidisciplinary management allowed successful breast-conserving treatment.

## Case presentation

In June 2025, a 57-year-old Iranian housewife, who had been postmenopausal for 3 years, without any past medical or surgical history, with a BMI of 33, and without any history of hormone replacement therapy, presented to our clinic accompanied by her daughter, who had previously been treated for invasive ductal carcinoma 2 years earlier and was attending a follow-up visit. The patient reported noticing a mass in her right breast. Physical examination revealed a palpable lesion in the upper outer quadrant of the right breast.

Bilateral mammography demonstrated an obscured 20 × 20 mm mass in the upper outer quadrant of the right breast, categorized as BI-RADS 0 (Supplemental Digital Content Figure S1, available at: https://links.lww.com/IJSCR/A169). Additionally, multiple bilateral partially circumscribed equal-density lesions were noted, along with diffuse punctate and cup-shaped calcifications, without evidence of architectural distortion, skin thickening, or retraction. No suspicious intramammary or axillary lymph nodes were identified. Complementary breast ultrasonography identified a 21.6 × 15.8 mm irregular hypoechoic mass with microcystic components and fine calcifications in the right breast, associated with dilated ducts containing internal septations and calcifications. Mild retroareolar ductal ectasia with avascular echogenic material was also present. In the left breast, multiple benign and indeterminate findings were present, including a complicated cyst measuring 8.1 × 5.4 mm, several simple cysts up to approximately 15 mm, and two clusters of microcystic areas measuring up to 32 × 15.9 mm and 17.4 × 7.8 mm. Mild retroareolar ductal ectasia with inspissated secretions was also noted. Overall, the ultrasound findings were categorized as BI-RADS IVa, and a few reactive lymph nodes with preserved morphology were observed bilaterally. In July, core needle biopsy of the right breast lesion demonstrated an infiltrative malignant epithelial neoplasm composed of cohesive nests, cords, and irregular glandular/tubular structures infiltrating a fibrotic stroma, consistent with invasive ductal carcinoma (IDC). The tumor cells exhibited moderate nuclear pleomorphism with enlarged hyperchromatic nuclei, irregular nuclear contours, conspicuous nucleoli, and readily identifiable mitotic figures. Overall histologic features were consistent with grade II IDC with an associated solid-type ductal carcinoma *in situ* component. No lymphovascular invasion, perineural invasion, or necrosis was identified (Fig. 1). Breast MRI further characterized the lesions, demonstrating in the right breast an irregular spiculated enhancing mass measuring 22 × 20 mm, corresponding to the known malignancy, along with a small additional 4.5 mm enhancing focus categorized as BI-RADS III. No evidence of chest wall invasion, nipple-areolar complex involvement, or definite suspicious axillary lymphadenopathy was identified. In the left breast, an irregular spiculated enhancing mass measuring 18 × 15 mm was observed in the central outer to posterior region with craniocaudal extension up to 25 mm, accompanied by adjacent non-mass enhancement measuring 22 × 16 mm, both highly suggestive of malignancy (BI-RADS V). No chest wall or nipple-areolar involvement was seen. TRU-CUT biopsy of the left-sided lesion confirmed invasive lobular carcinoma (ILC) with a Nottingham grade II/III, with lobular carcinoma in situ also present. Lymphovascular invasion was not identified (Fig. 2).

**Figure 1.**
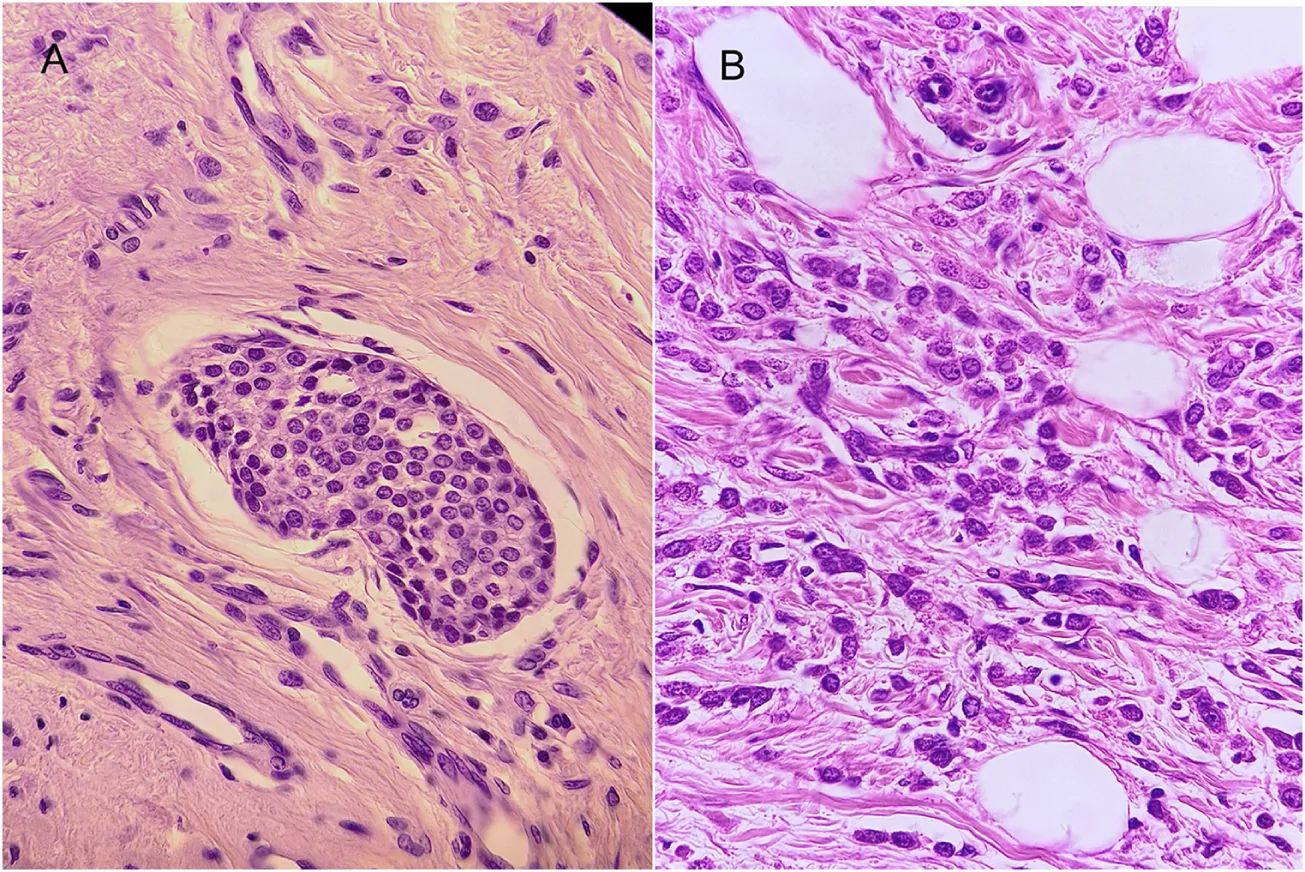
H&E 400× of ILC: (A) Sections show a discohesive infiltrative neoplastic proliferation composed of small uniform round-to-oval tumor cells with mild to moderate atypia. (B) The cells infiltrate the stroma in single-file and occasional targetoid patterns around ducts, vessels, and adipose tissue. Mitotic figures are infrequent, and adjacent lobular units are expanded by discohesive neoplastic cells, consistent with LCIS.

**Figure 2.**
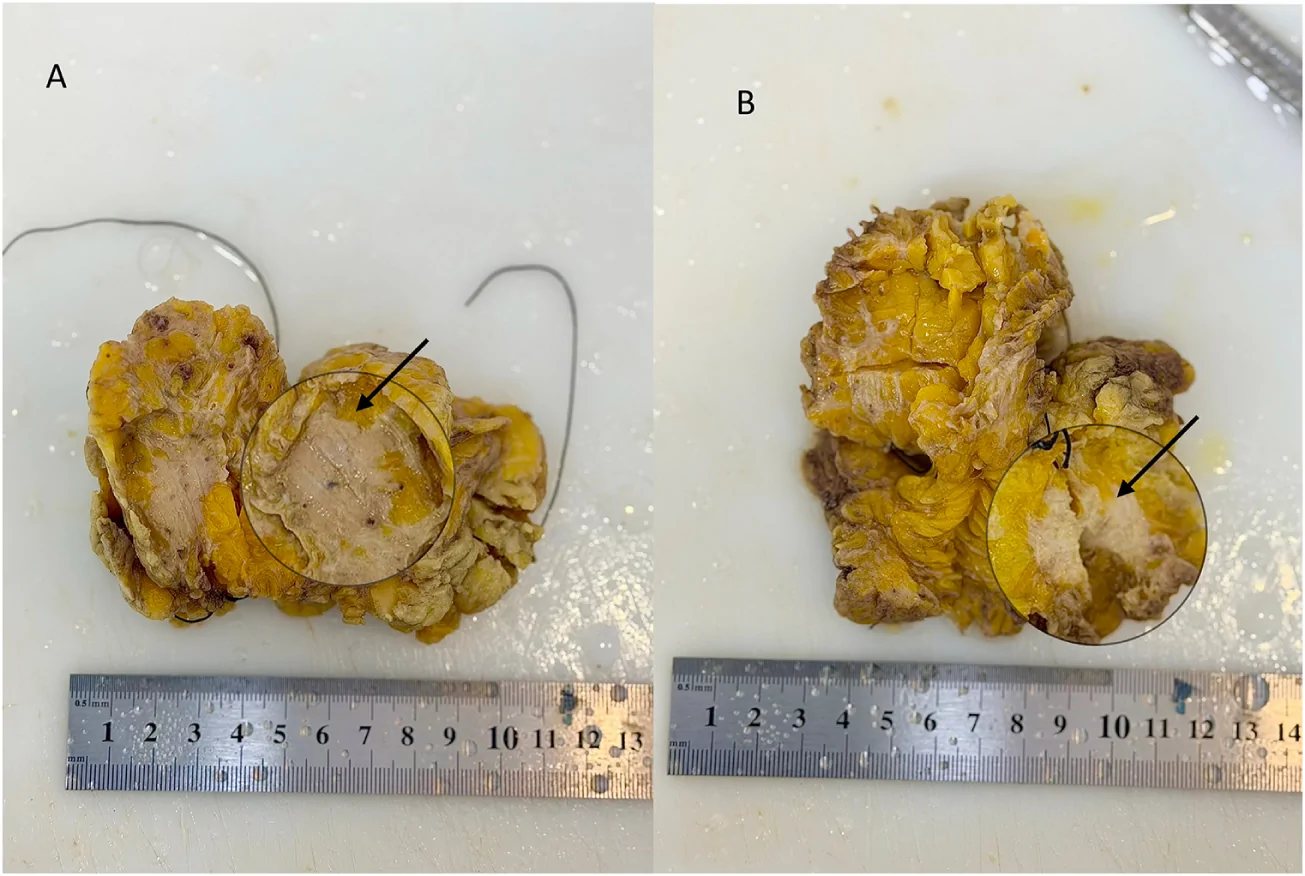
(A) shows the gross view of IDC, and (B) shows the gross view of ILC.

In September 2025, the patient underwent breast-conserving surgery, including bilateral lumpectomy and axillary lymph node dissection. Gross examination of the surgical specimens demonstrated distinct tumor lesions in both breasts, with the right-sided tumor (Fig. 2A) measuring approximately 10.6 × 7.4 mm and the left-sided tumor (Fig. 2B) measuring approximately 9.5 × 8.6 mm. Histopathologic examination showed distinct neoplasms in each breast. The right breast specimen demonstrated invasive and in situ ductal carcinoma, with moderate estrogen receptor positivity (60–70%), negative progesterone receptor expression, HER2 negativity (1+), a Ki-67 index of 25–27%, and preserved E-cadherin (Fig. 3A). The left breast specimen revealed invasive and in situ lobular carcinoma characterized by strong estrogen (90%) and progesterone receptor (70%) positivity, HER2 negativity (0+), low proliferative activity (Ki-67 5%), and loss of E-cadherin expression (Fig. 3B); the grading of these specimens was similar to TRU-CUT biopsy. The patient has been undergoing routine radiotherapy and letrozole as adjuvant therapy since September 2025 and has remained symptom-free and satisfied with the results of therapy. The timeline of the patient’s clinical presentation, diagnostic workup, surgical management, and postoperative histopathologic evaluation is presented in Table 1.

**Figure 3.**
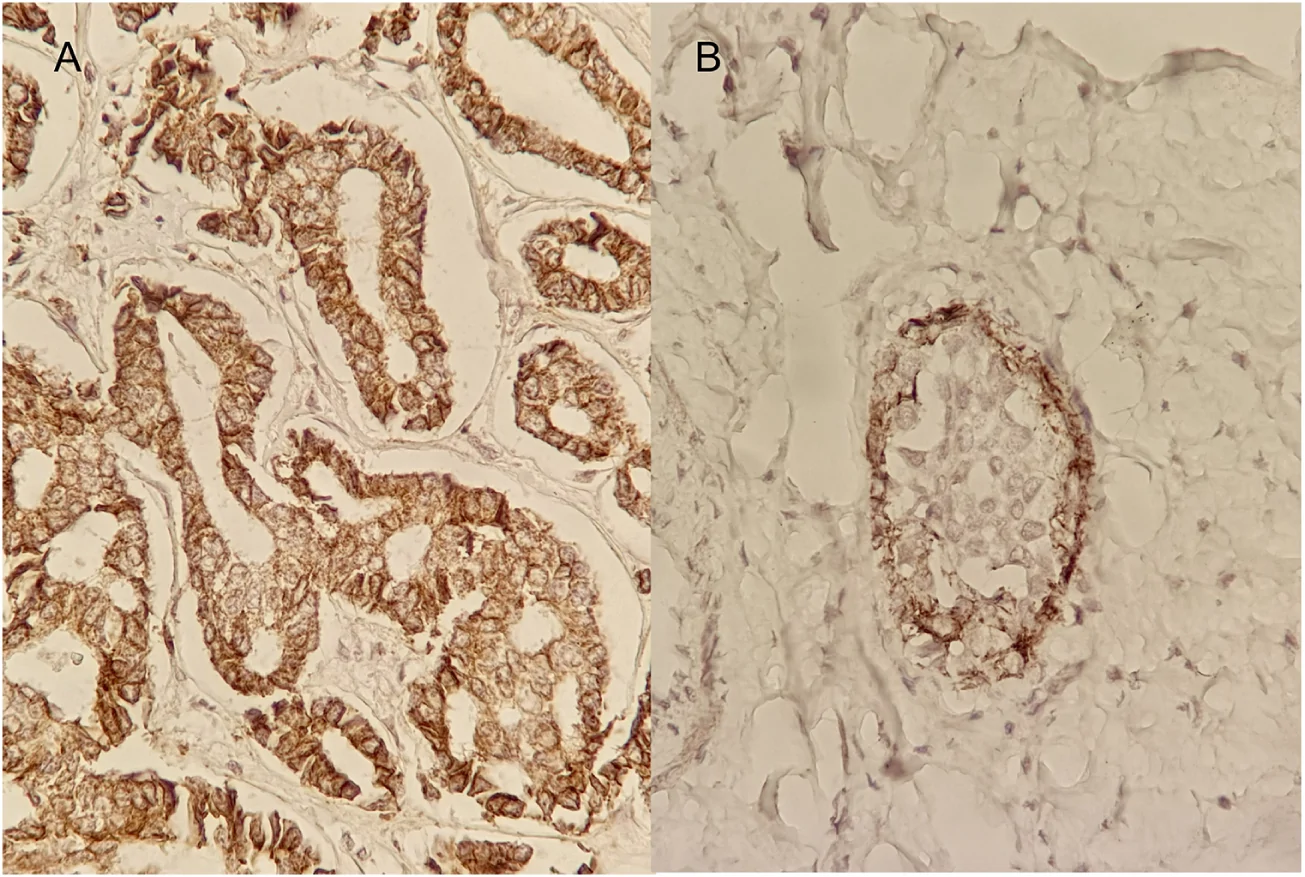
IHC, 400×: (A) Neoplastic epithelial cells show preserved membranous E-cadherin expression, supporting a ductal phenotype (IDC). (B) Neoplastic cells show loss of membranous E-cadherin expression, consistent with a lobular phenotype (ILC).

**Table 1:** Timeline of the patient’s clinical presentation, diagnostic workup, surgical management, and postoperative histopathologic evaluation for synchronous bilateral primary breast carcinoma.

Date	Clinical event
15 July 2025	Bilateral mammography performed, demonstrating an obscured right breast mass with additional bilateral benign-appearing findings.
18 July 2025	Complementary breast ultrasonography performed, confirming a suspicious right breast lesion and additional bilateral cystic findings.
29 July 2025	Core needle biopsy of the right breast lesion performed.
14 September 2025	Breast MRI performed, further characterizing the right breast malignancy and identifying suspicious lesions in the left breast.
29 September 2025	Core needle biopsy of the left breast lesion confirmed invasive lobular carcinoma.
29 September 2025	Bilateral breast-conserving surgery with lumpectomy and axillary lymph node dissection performed; surgical pathology completed.
14 October 2025	Immunohistochemical analysis performed on both surgical specimens.
September 2025 onward	Routine adjuvant radiotherapy initiated; the patient remained symptom-free on follow-up.

### Patients’ perspective

The patient expressed concern regarding the implications of her diagnosis but was relieved that the disease was detected incidentally at an early stage. She reported that her daughter, who had previously experienced a similar illness, provided substantial emotional and practical support throughout her treatment, and she expressed hope that more definitive treatments for cancer would become available in the near future.

## Discussion

This case describes a 57-year-old woman diagnosed with synchronous bilateral breast cancer presenting with distinct histologic subtypes in each breast. Initial evaluation of a palpable right breast mass revealed IDC, while further imaging identified an occult contralateral lesion, subsequently confirmed as ILC. The simultaneous presence of invasive ductal and lobular carcinomas underscores the biological heterogeneity of bilateral breast cancer and highlights the diagnostic value of comprehensive bilateral imaging. This case emphasizes the importance of thorough contralateral breast assessment and individualized multidisciplinary management in patients with synchronous bilateral disease.

ILCs exhibit distinct clinicopathological features, including a propensity for larger tumor size, multifocality, indolent growth, and frequent mammographic undetectability^[^5^]^.

Population-based studies show that synchronous and metachronous bilateral breast cancers are biologically and prognostically distinct. Synchronous disease mirrors unilateral incidence patterns, while metachronous tumors are more common in younger patients and have declined with widespread adjuvant therapy. Improved imaging, including mammography and MRI, allows earlier detection of contralateral tumors, as illustrated in this case, where comprehensive imaging identified histologically distinct bilateral malignancies^[^6^]^.

IDC and ILC are biologically and pathologically distinct. ILC is often larger, lower grade, and strongly hormone receptor–positive, with low HER2 expression, but is frequently diagnosed at a more advanced stage with greater nodal involvement due to its diffuse growth and subtle imaging features. Its indolent behavior and risk of late recurrence further distinguish it from IDC, reflecting inherent differences in tumor biology^[^7^]^.

IDC typically appears as a discrete spiculated mass with or without calcifications on conventional imaging, facilitating earlier detection, whereas ILC often demonstrates subtle or occult findings due to its diffuse infiltrative growth pattern. ILC may present as architectural distortion, asymmetry, or non-mass lesions, leading to underestimation of tumor extent and delayed diagnosis, particularly on mammography. Breast magnetic resonance imaging offers improved sensitivity for ILC by better delineating tumor extent and detecting multifocal or contralateral disease, highlighting its role in the evaluation of suspected lobular carcinoma^[^8^]^.

The surgical management of synchronous bilateral breast cancer requires an individualized strategy that ensures oncologic adequacy while minimizing treatment burden. Although bilateral mastectomy has historically been favored because of concerns regarding multifocality and local control, contemporary evidence supports breast-conserving surgery in carefully selected patients when clear margins and adjuvant radiotherapy are feasible. Recent surgical series have shown comparable oncologic outcomes between breast-conserving approaches and mastectomy in early-stage bilateral disease, emphasizing the importance of tumor biology, extent of disease, and patient preference in surgical decision-making. In this case, bilateral breast-conserving surgery with axillary evaluation was consistent with current evidence-based practice^[^9,10^]^.

Synchronous bilateral breast cancer has been associated with less favorable disease-free survival compared with metachronous disease, while overall prognosis is largely driven by tumor stage and hormone receptor status rather than bilaterality alone. Hormone receptor positivity has been identified as a favorable prognostic factor that may offset the adverse impact of synchronous presentation. Accordingly, the hormone receptor–positive profile observed in this case may be associated with a more favorable prognosis despite bilateral disease^[^11^]^.

This case illustrates the importance of comprehensive imaging and independent pathological assessment in synchronous bilateral breast cancer. The coexistence of ductal and lobular carcinomas highlights tumor heterogeneity, underscoring the need for tailored management of each lesion. Early detection enabled successful breast-conserving surgery with axillary staging, and receptor profiling guided adjuvant therapy. Clinicians should maintain a high index of suspicion in high-risk patients and individualize treatment plans to optimize outcomes while preserving quality of life.

This report is limited by its nature as a single case, which restricts generalizability to broader populations. Long-term outcomes and recurrence risk cannot yet be assessed, as follow-up is ongoing. Additionally, molecular profiling beyond standard receptor status was not performed, which may have provided further insights into tumor biology and potential therapeutic targets. Despite these limitations, the case offers valuable lessons on the diagnosis and management of synchronous bilateral breast cancer with distinct histologic subtypes.

## Conclusion

This case illustrates the heterogeneity of synchronous bilateral breast cancer, with coexisting ductal and lobular carcinomas. Comprehensive imaging and independent pathological assessment enabled timely diagnosis and guided breast-conserving surgery with adjuvant therapy. It emphasizes the importance of individualized, multidisciplinary management to optimize outcomes in such patients.

## Data Availability

Data supporting this study’s findings were extracted from the patient’s medical records using a structured data collection form. Relevant clinical information, imaging findings, and histopathological details may be obtained from the corresponding author upon reasonable request, subject to institutional policies and patient privacy requirements.
